# Glycation of alpha-synuclein enhances aggregation and neuroinflammatory responses

**DOI:** 10.1038/s41531-025-01159-w

**Published:** 2025-10-23

**Authors:** Eftychia Vasili, Annekatrin König, Mohammed Al-Azzani, Clara Bosbach, Luisa Maria Gatzemeier, Searlait Thom, Ana Chegão, Hugo Vicente Miranda, Claudia Steinem, Daniel Erskine, Tiago F. Outeiro

**Affiliations:** 1 University Medical Center Gottingen, Department of Experimental Neurodegeneration, Center for Biostructural Imaging of Neurodegeneration, Göttingen, Germany; 2 Institute of Organic and Biomolecular Chemistry, Georg-August-University Göttingen, Göttingen, Germany; 3 Translational and Clinical Research Institute, Faculty of Medical Sciences, Newcastle University, Newcastle upon Tyne, UK; 4 iNOVA4Health, NOVA Medical School, NMS, Universidade NOVA de Lisboa, Lisboa, Portugal; 5 Max Planck Institute for Multidisciplinary Sciences, Göttingen, Germany; 6 Scientific employee with an honorary contract at Deutsches Zentrum für Neurodegenerative Erkrankungen (DZNE), Göttingen, Germany

**Keywords:** Biochemistry, Diseases, Neurology, Neuroscience

## Abstract

The risk of developing Parkinson’s disease (PD) is elevated in individuals with type 2 diabetes (T2DM), but the molecular pathways underlying this link remain unclear. Glycation, a non-enzymatic modification of lysine and arginine residues by reducing sugars or reactive dicarbonyls, may disrupt proteostasis and trigger pathology. Here, we investigated how methylglyoxal (MGO)- and ribose-mediated glycation influence aSyn aggregation, neuroinflammation, and detoxification pathways. Using SH-SY5Y cells, primary neurons, primary microglia and MGO-injected aSyn transgenic mice, we found that MGO-glycated aSyn promotes PD associated pathological features, including pS129-positive aSyn aggregates, neuroinflammation, and impairment of the glyoxalase detoxification pathway. Ribose-glycated aSyn, while immunogenic, exerts limited effects on aggregation and seeding. Both glycated species activates microglia and upregulate pro-inflammatory markers. We further developed a novel antibody specific for MGO-glycated aSyn, which selectively detects Lewy body–like deposits in dementia with Lewy bodies (DLB) tissue and MGO-injected mice. These findings implicate MGO-glycation in PD-T2DM comorbidity.

## Introduction

Glycation, also referred to as non-enzymatic glycosylation, or Maillard reaction, is the reaction between reducing sugars or other aldehydes and amino groups of proteins, nucleotides, or lipids^1^. Glycation is actually a complex network of different reactions, that involve the formation of Schiff bases in the first steps^2^. Early glycation products, also called Amadori compounds, are formed via condensation of a carbohydrate form with a primary amino group of amino acids, nucleic acids, or amino lipids. The Amadori compound then slowly degrades through different chemical pathways yielding either advanced glycation end products (AGEs) or highly reactive dicarbonyls, such as methylglyoxal (MGO)^3^. Finally, higher and irreversible molecular weight species (advanced glycation end products—AGEs) are generated^4^. Proteins and peptides can be modified at the N-terminus, at arginine or lysine side chains, or at cysteine side chains, especially by dicarbonyls. However, since glycation is a non-enzymatic reaction, it is not limited to defined residues or specific physiological conditions. Although several reducing sugars, such as glucose, fructose, and ribose, can contribute to glycation processes, MGO is considered the most efficient glycating agent in vivo. MGO is formed as a side product in glycolysis^5–7^ and from the catabolism of amino acids, and can diffuse extracellularly, enabling glycation of both intracellular and extracellular proteins. Dicarbonyl compounds like MGO are estimated to be approximately 200–50,000-fold more reactive towards amino groups than glucose^8^. As a result, MGO can rapidly and extensively modify proteins even at the micromolar or nanomolar concentrations typically observed in physiological settings^9,10^.

Alpha-synuclein (aSyn), a central player in the etiology and progression of Parkinson´s disease (PD) and related disorders (collectively known as synucleinopathies) is, like many other proteins, subject to glycation reactions^11^. However, given that aSyn is rich in lysines (15 residues in its primary amino acid sequence), and particularly long-lived, aSyn-AGEs can form and accumulate^12^. We and others have previously shown that glycation affects several aspects of aSyn biology. In particular, glycation impairs aSyn clearance due to interference with ubiquitination and SUMOylation of lysine residues, reduces extracellular protease cleavage, and impairs membrane binding^13–15^. MGO-mediated glycation was shown to affect mainly the lysine residues in the N-terminal region of aSyn^13^. Interestingly, glycation affects aSyn aggregation kinetics, enhancing oligomerization and reducing fibrilization^11–13,16–19^. Around five MGO-glycated residues per aSyn molecule were shown to optimally inhibit aSyn aggregate elongation, reducing the incorporation of further aSyn monomers into the fibrils^11^. Strikingly, intracerebroventricular injection of MGO aggravates motor, cognitive, olfactory, and colonic dysfunction in a mouse model of synucleinopathy^20^.

The metabolic disease diabetes mellitus type 2 (T2DM), characterized by sugar dyshomeostasis and increased levels of protein glycation, is a known risk factor for PD^21–24^. Given the particular vulnerability of neurons to high glucose levels, glycation, and associated oxidative stress, understanding the effects of glycation in proteins of interest in the context of brain disorders is essential. Apart from the direct consequences of hyperglycemia that are thought to account for most of the T2DM associated complications, higher glucose levels also cause increased production of reactive oxoaldehydes such as MGO^6,25^. In addition, abnormally high levels of D-ribose, an important contributor to protein glycation, are detected in the urine of T2DM patients^26^. Thus, in this study, we investigated the molecular effects of these two agents (MGO and ribose) on aSyn spreading, seeding, and neuroinflammation. In addition, we developed novel aSyn glycation-specific polyclonal antibodies that will boost our understanding of the effects of aSyn glycation in PD and in other synucleinopathies. Ultimately, we anticipate that our findings may open novel perspectives for therapeutic intervention.

## Results

### Ribose- and MGO-glycation differently affect aSyn pathology

In a previous study, we investigated the aggregation of aSyn modified by eight different glycating agents. Among these, MGO and ribose were identified as the most efficient, modifying aSyn to the greatest extent. Both MGO-modified aSyn (aSyn-MGO) and ribose-modified aSyn (aSyn-ribose) were found to inhibit fibril elongation while leaving nucleation largely unaffected. This inhibition leads to the accumulation of intermediate species—referred to as “paucimers” (from the Latin paucus, meaning “few”)—which are smaller, soluble aSyn oligomers that remain in the soluble fraction and are not incorporated into mature fibrils, as previously characterized^11^. Both glycated forms reduce binding of aSyn to membranes and suppress the formation of ThT-positive fibrils. Notably, MGO demonstrated significantly higher reactivity than ribose, with an estimated potency 30–50 times greater^11^. Therefore, in the present study, we investigated the seeding effects of aSyn-MGO and aSyn-ribose on various cellular models. First, we assessed the effects of aSyn-MGO and aSyn-ribose glycated species-generated as we previously described^11^, in SH-SY5Y cells conditionally expressing aSyn^27^. SH-SY5Y cells were differentiated into neuron-like cells using retinoic acid and, on day 4 of differentiation, we exogenously added 100 nM of either glycated or non-glycated aSyn. The cells were then incubated for an additional 4 days before further analysis (Supplementary Fig. 1A). Double immunostaining for Tuj1, a neuron-specific marker, and total aSyn was performed at day 8 and confirmed the induction of the neuronal marker, confirming the neuronal phenotype, and the formation of aSyn deposits. Despite the many open questions regarding the precise role of aSyn in PD pathology, the formation of aggregates containing insoluble aSyn phosphorylated on serine 129 (pS129) is an important hallmark of PD and other synucleinopathies. Co-staining with Tuj1 and p-aSyn on Ser129 revealed the presence of phosphorylated aggregates particularly following treatment with aSyn and aSyn-MGO (Supplementary Fig. 1B, C). However, treatment with aSyn-ribose resulted in no obvious increase of pS129-aSyn. Notably, we observed that S129-positive aggregates colocalize with the nucleus. This is consistent with our recent findings^28^, and with previous studies showing that MGO-mediated glycation of aSyn alters its localization by reducing membrane binding, likely due to modifications in its N-terminal region. These changes impair aSyn clearance and promote the accumulation of toxic oligomers, which may contribute to disrupted cellular function and neurodegenerative processes^13^.

**Fig. 1:**
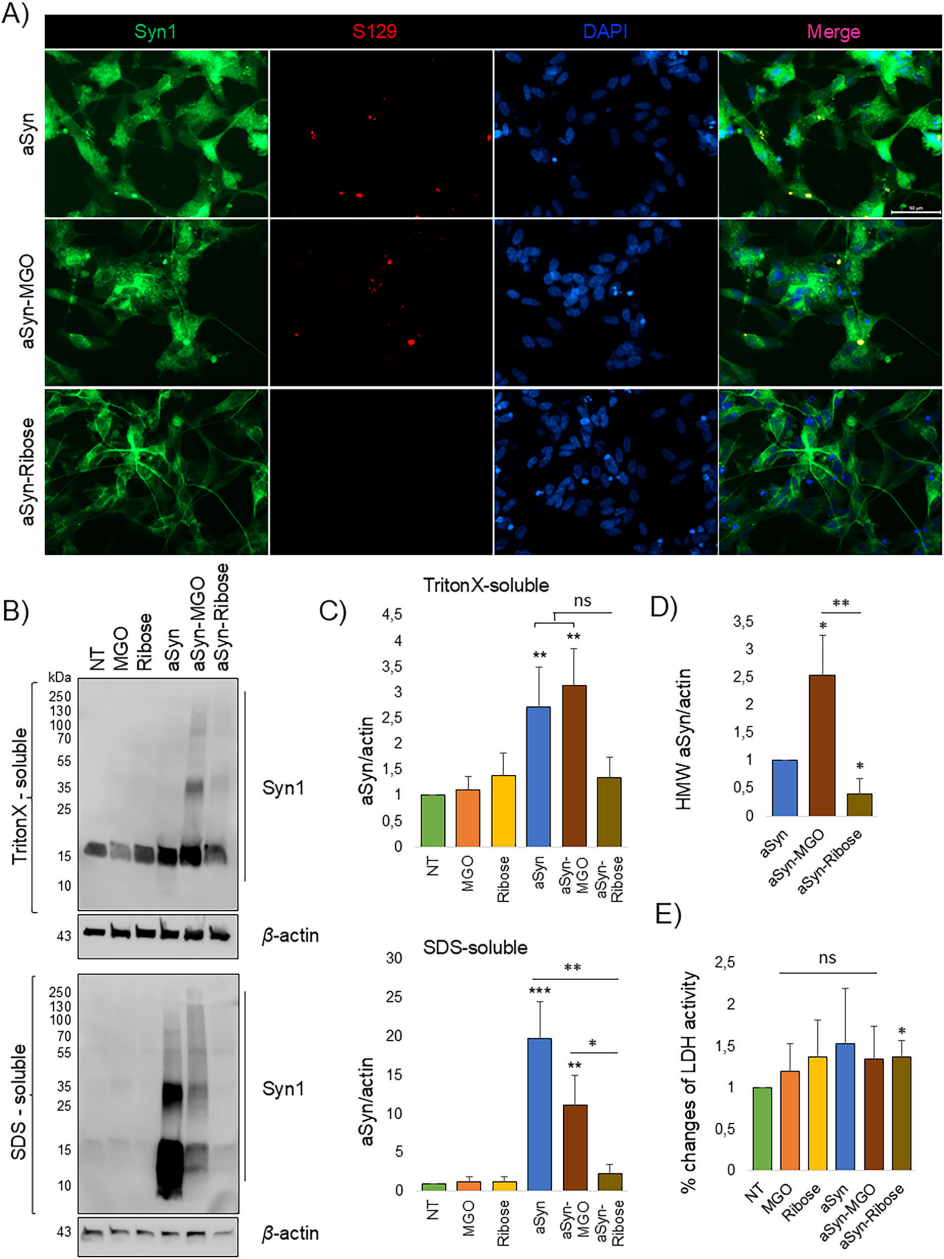
MGO-glycated aSyn induces the formation of pS129 positive inclusions in differentiated SH-SY5Y cells. **A** SH-SY5Y cells, conditionally expressing aSyn under a Tet-Off cassette were differentiated to neuron-like cells, expressing human wildtype aSyn (doxycycline was omitted from the medium). aSyn, that had been incubated with or without glycating agent was added to the cells at a final concentration of 100 nM for 4 days. Cells were immunostained using Syn1 and pS129, representative images from *N* = 3 (Scale bar 50 μm). **B** Cells were sequentially extracted with TritonX-100 and SDS containing buffer and analyzed using immunoblots with Syn1 antibodies. β-Actin was used a loading control and **C** the ratio of aSyn versus β-actin was quantified (*n* = 3, mean ± SD). **D** The ratio of aSyn HMWs (above ~55 kDa) versus β-actin was quantified (*n* = 3, mean ± SD). **E** Cytotoxicity under different conditions of treatment was measured by the activity of released LDH and is displayed as per cent cytotoxicity (*n* = 4).

As the glycation protocol involved gentle shaking at 37 °C, possibly causing the formation of fibrils and oligomers, it was expected that these control aSyn preparations would lead to the formation of pS129-positive aggregates (Fig. 1A). Correspondingly, in order to further assess aSyn seeding under the treatment with the three different aSyn proteins, we performed a biochemical assay well described previously^29^ based on detergent fractionation of soluble and insoluble aSyn. Western blotting of the Triton X-100-soluble fraction using the Syn-1 antibody showed a significant increase of monomeric aSyn under treatment with each of the three different aSyn proteins. In the SDS-soluble fraction, aSyn migrated at higher molecular weights (HMW) especially under treatment with aSyn and aSyn-MGO, with a molecular weight (MW) ranging from 25 to 250 kDa on SDS-PAGE (Fig. 1B, C). Quantification of the high molecular weight (HMW) species (~up to 55 kDa) in the SDS-soluble fraction revealed a clear increase in these species following aSyn-MGO treatment, with levels approximately 1.5-fold higher compared to unmodified aSyn treatment (Fig. 1D). In contrast, aSyn-ribose treatment did not result in a significant increase in HMW species. In addition, treatment with either glycating agent or aSyn alone did not cause significant cytotoxicity, although a non-significant increasing trend was observed. However, a slight but measurable increase in LDH levels was observed only in cells treated with aSyn-ribose, while aSyn-MGO had no significant effect compared to controls (Fig. 1E).

**Fig. 2:**
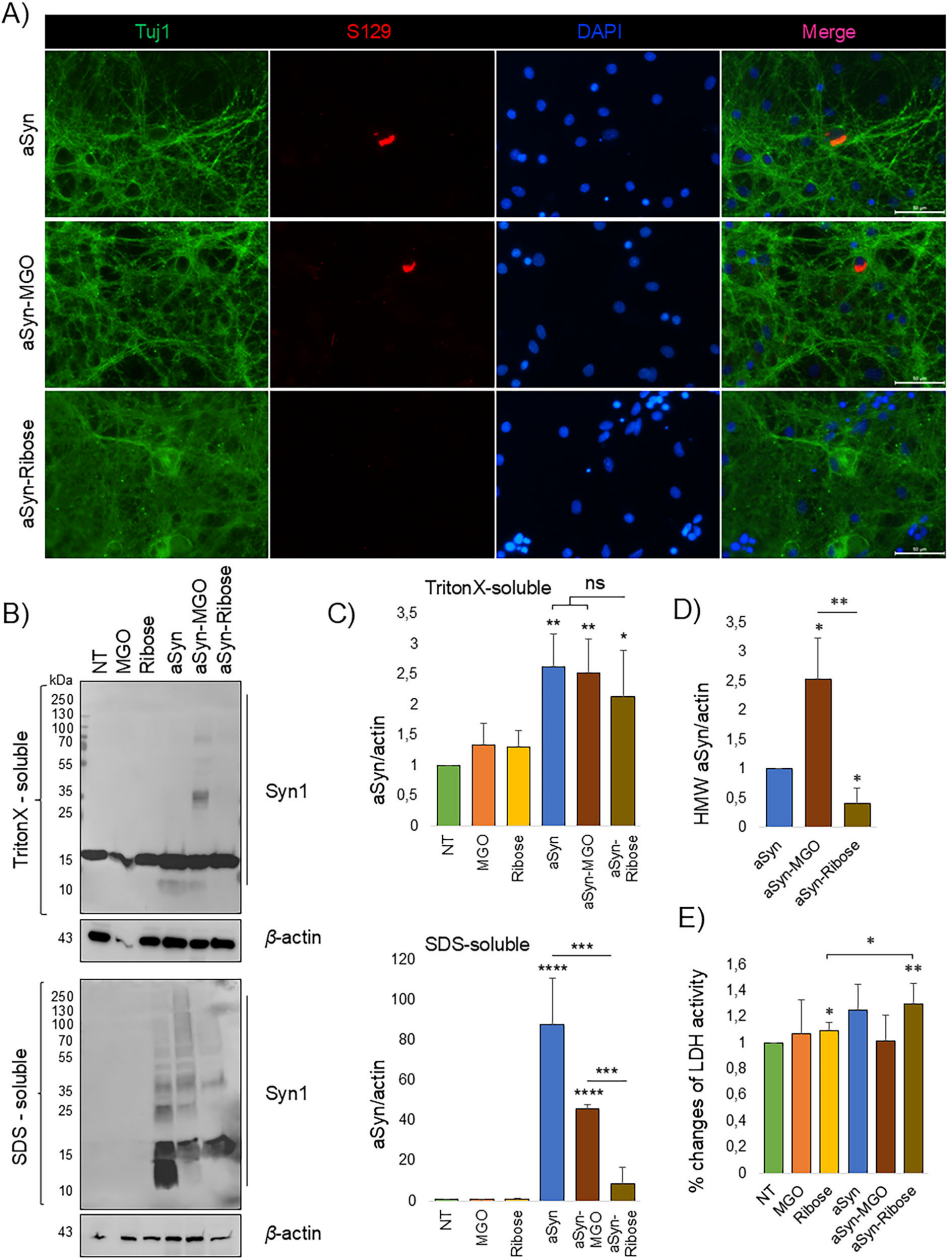
MGO-glycated aSyn induces pS129 positive inclusions in primary neurons. **A** Primary rat cortical neurons were incubated for 20 days with 50 nM aSyn species. Following wash and fixation, cells were immunostained with Tuj1 and pS129, representative images from *N* = 3 (Scale bar 50 μm). **B** After sequentially extracting the cells with TritonX-100 and SDS containing buffer, extracts were analyzed using immunoblots with Syn1 and β-actin as loading control. **C** The ratio of aSyn and β-actin signal was determined (*n* = 4, mean ± SD). **D** The ratio of aSyn HMWs (above ~55 kDa) versus β-actin was quantified (*n* = 3, mean ± SD). **E** Cytotoxicity under different conditions of treatment was measured by the activity of released LDH and is displayed as per cent cytotoxicity (*n* = 5).

Next, we performed similar experiments in cortical neuronal cultures from wild type rats. At DIV5, we exogenously added the glycated proteins at a concentration of 50 nM and further incubated for 20 days. Immunostaining for pS129-aSyn demonstrated the formation of large phosphorylated aSyn inclusions in neuronal cultures incubated with aSyn or with aSyn-MGO proteins (Fig. 2A). In agreement with the results obtained in SH-SY5Y cells, we detected no phosphorylated aSyn aggregates upon treatment with aSyn-ribose. Quantification of cellular assays revealed a significant increase in pSer129-positive inclusions following treatment with glycated aSyn with MGO compared to unmodified aSyn. In contrast, no increase in pSer129 aggregation was observed in cells treated with ribose-modified aSyn (Supplementary Fig. 2A). As a control we treated neuronal cultures with aSyn pre-formed fibrils (PFFs, Supplementary Fig. 2B, C). Next, we performed Western blotting analysis primary neuronal cultures, and observed a significant increase of aSyn HMW species upon treatment with aSyn or aSyn-MGO in the SDS-soluble fraction. Treatment with aSyn-ribose caused a small, yet statistically significant increase in detergent-insoluble aSyn species (Fig. 2B–D), indicating that the effect is real but relatively minor compared to other treatment conditions. HMW species were absent in non-treated cells (NT) and cells treated with only MGO or ribose. Interestingly, we observed the same cytotoxic effect of aSyn-ribose in cortical neuronal cultures (Fig. 2E), which may reflect the formation of partially toxic oligomeric intermediates that cause mild membrane damage without inducing robust aggregation or severe cytotoxicity.

Overall, these data demonstrate a distinct seeding behavior between aSyn-MGO and aSyn-ribose, suggesting that the nature of the glycation modification influences the ability of aSyn species to induce aggregation. Specifically, aSyn-MGO exhibits a greater propensity to induce the accumulation of HMW aggregates, whereas aSyn-ribose shows minimal seeding activity and does not induce the formation of pS129 aggregates, indicating fundamental differences in the aggregation and in the pathological potential of these glycated species.

### Ribose- and MGO-glycation decrease Glo I activity

Although AGEs occur spontaneously, cells possess active mechanisms to counteract glycation. A key detoxification pathway involves the glyoxalase system—comprising Glyoxalase I (Glo I), Glyoxalase II, and aldose reductases^30,31^. Notably, Glo I levels decline with age in the human brain^32^, and are reduced in the substantia nigra of PD patients^33^ and in PD models^34^. Additionally, aSyn deficiency in mice alters AGE levels and increases both Glo I expression and glycation stress^35^, highlighting a role for aSyn in sugar metabolism. Consistently, we found that MGO-induced cytotoxicity was abolished by aSyn knockdown in iPSCs, reinforcing the link between aSyn levels and glycation-related toxicity^13^.

**Fig. 3:**
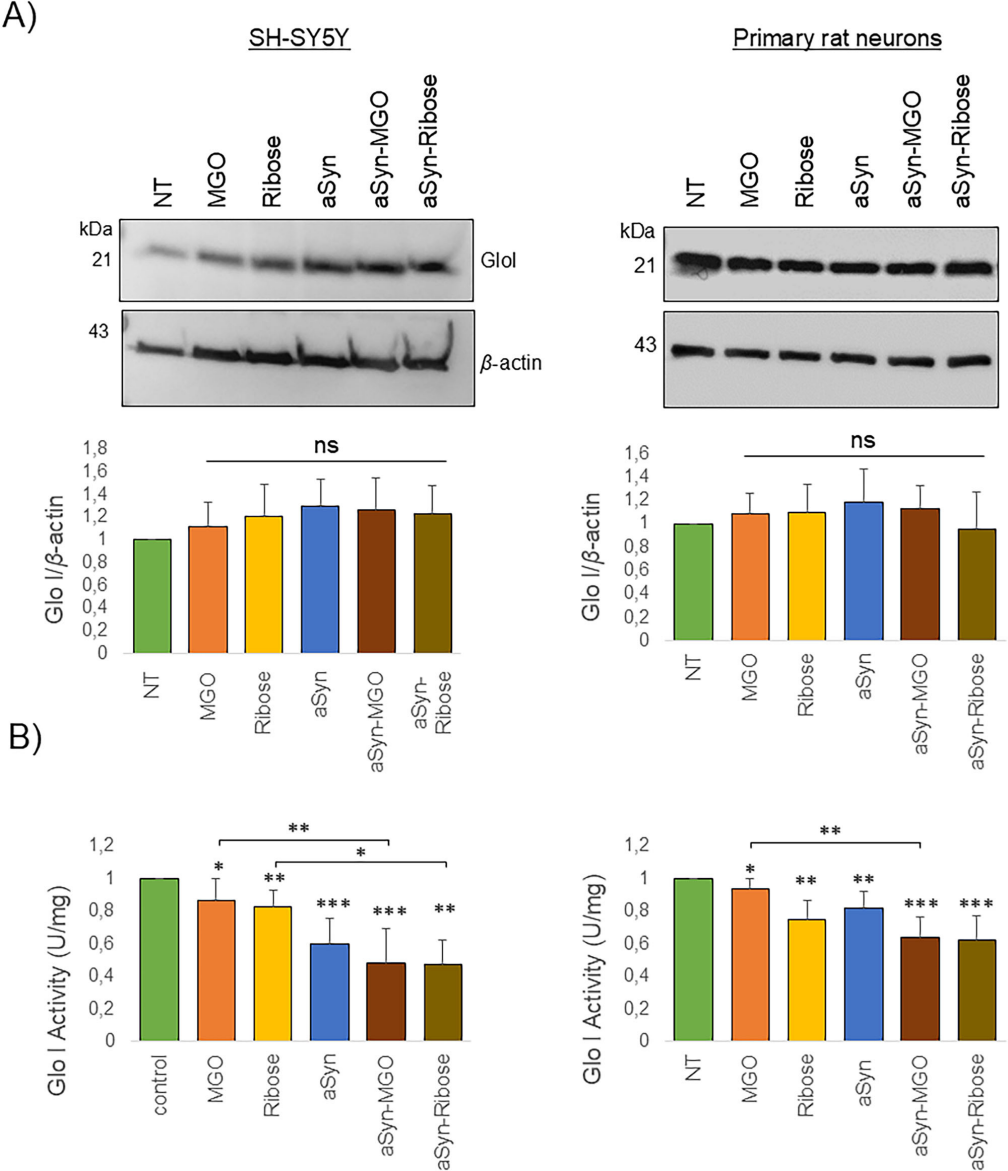
aSyn glycation decrease Glo I activity. **A** Protein extracts were analyzed using immunoblots with Glo I and β-actin as loading control. The ratio of Glo I and β-actin signal was determined (*n* = 4, mean ± SD). **B** Determination of Glo I activity in SH-SY5Y (i) and primary neuronal cultures (ii) cell lysates. All measurements were performed following kit protocol (*n* = 5, mean ± SD).

To assess whether exposure to glycated aSyn (aSyn-MGO and aSyn-ribose) disrupts the glyoxalase system in SH-SY5Y cells and primary neurons, we measured both Glo I protein levels and enzymatic activity in the same samples. While the levels of Glo I did not change after treatment with glycated aSyn (Fig. 3A), the enzymatic activity of Glo I was significantly reduced, especially following treatment with aSyn-MGO and aSyn-ribose (Fig. 3B), suggesting impaired function of the glyoxalase pathway.

### Glycated aSyn induces neuroinflammatory responses in microglial cells

Chronic microglia activation and upregulation of proinflammatory factors are typical hallmarks of neurodegenerative processes, creating a vicious circle in which neurodegeneration and neuroinflammation fuel each other^36^. Disruption of the glyoxalase pathway can contribute to an inflammatory environment, thereby promoting pathogenesis in neurodegenerative disease^37,38^.

**Fig. 4:**
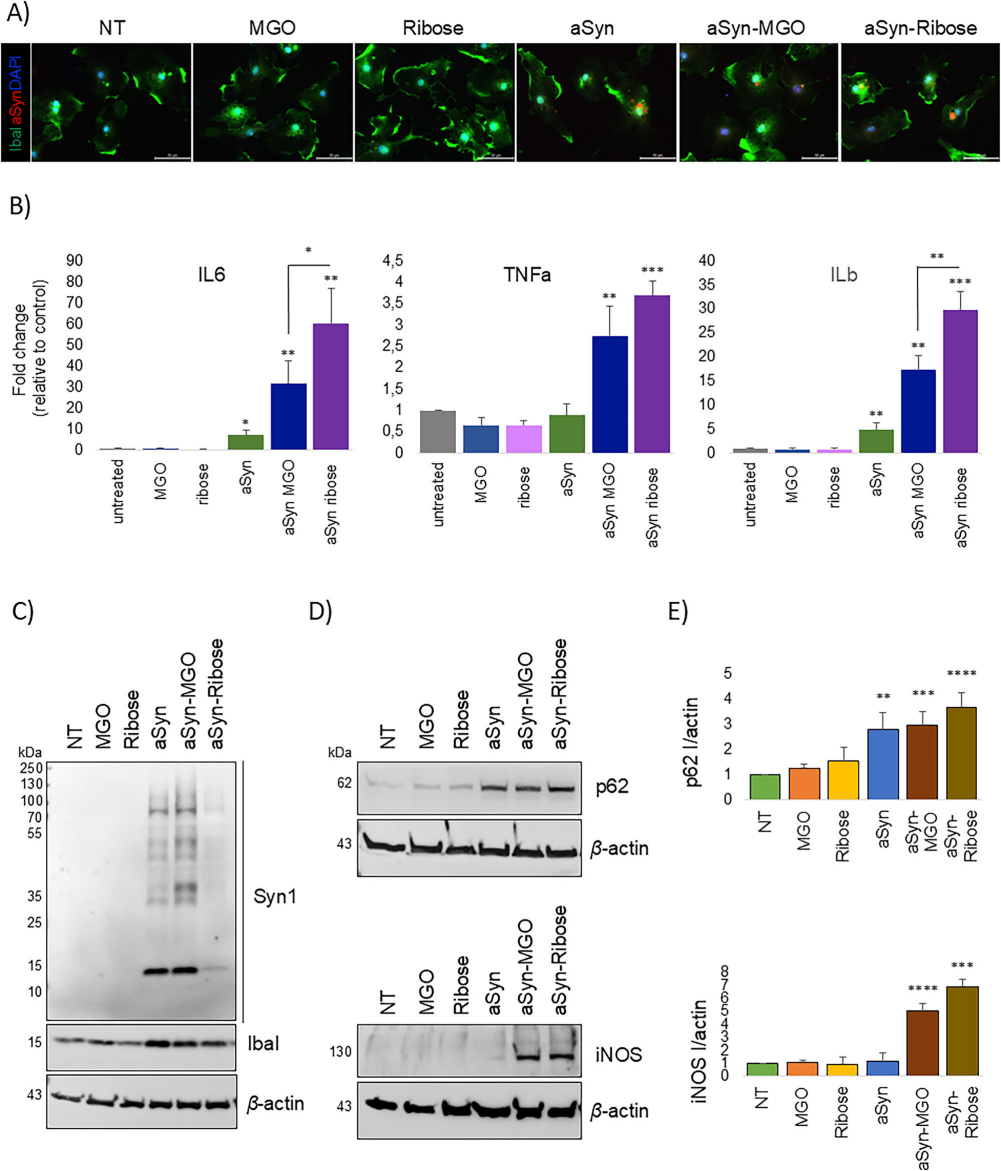
aSyn glycation induces neuroinflammatory responses in microglia. **A** Representative immunofluorescence images of microglia treated with 100 nM aSyn species for 24 h, stained with Iba1 (green) and Syn1 (red) antibodies (*n* = 3, Scale bar 50 μm). **B** Quantitative PCR analysis of pro-inflammatory genes (IL-6, TNF-α, IL-1β) in microglia treated with aSyn species; β-actin was used for normalization (*n* = 3). **C** Immunoblotting of RIPA protein extracts from treated microglia, probed with Syn1 and *β*-actin antibodies. **D** Immunoblot analysis of Iba1, p62, and iNOS expression in the same samples; *β*-actin served as loading control. **E** Densitometric quantification of p62 and iNOS signal intensity (*n* = 4; mean ± SD).

Using ionized calcium-binding adapter molecule 1 (Iba1) as a marker of cells of the myeloid lineage including microglia, we confirmed the uptake of glycated aSyn (Fig. 4A). In addition, we found that treatment with glycated aSyn led to a significant increase in the production of proinflammatory cytokines IL6, TNF-α and IL-1β (Fig. 4B). Consistently, inducible Nitric Oxide Synthase (iNOS), an enzyme upregulated in response to inflammatory stimuli, and p62, a multi-functional adaptor protein involved in various cellular processes, were elevated following incubation with both ribose- and MGO-glycated aSyn (Fig. 4C–E).

Although both glycated aSyn species induced neuroinflammatory responses, they exhibited distinct uptake profiles by microglia. In particular, aSyn-ribose was uptaken to a lesser extent when compared to aSyn-MGO (Fig. 4C, Supplementary Fig. 3). Using the LDH assay as a readout, we found no differences in cytotoxicity under all tested conditions (Supplementary Fig. 3). Interestingly, while both ribose- and MGO-glycated aSyn triggered neuroinflammation, they differentially influenced the accumulation of pS129 aSyn in SH-SY5Y and primary neurons, suggesting divergent mechanisms in their contribution to pathology.

### A novel polyclonal antibody detects glycated aSyn

**Fig. 5:**
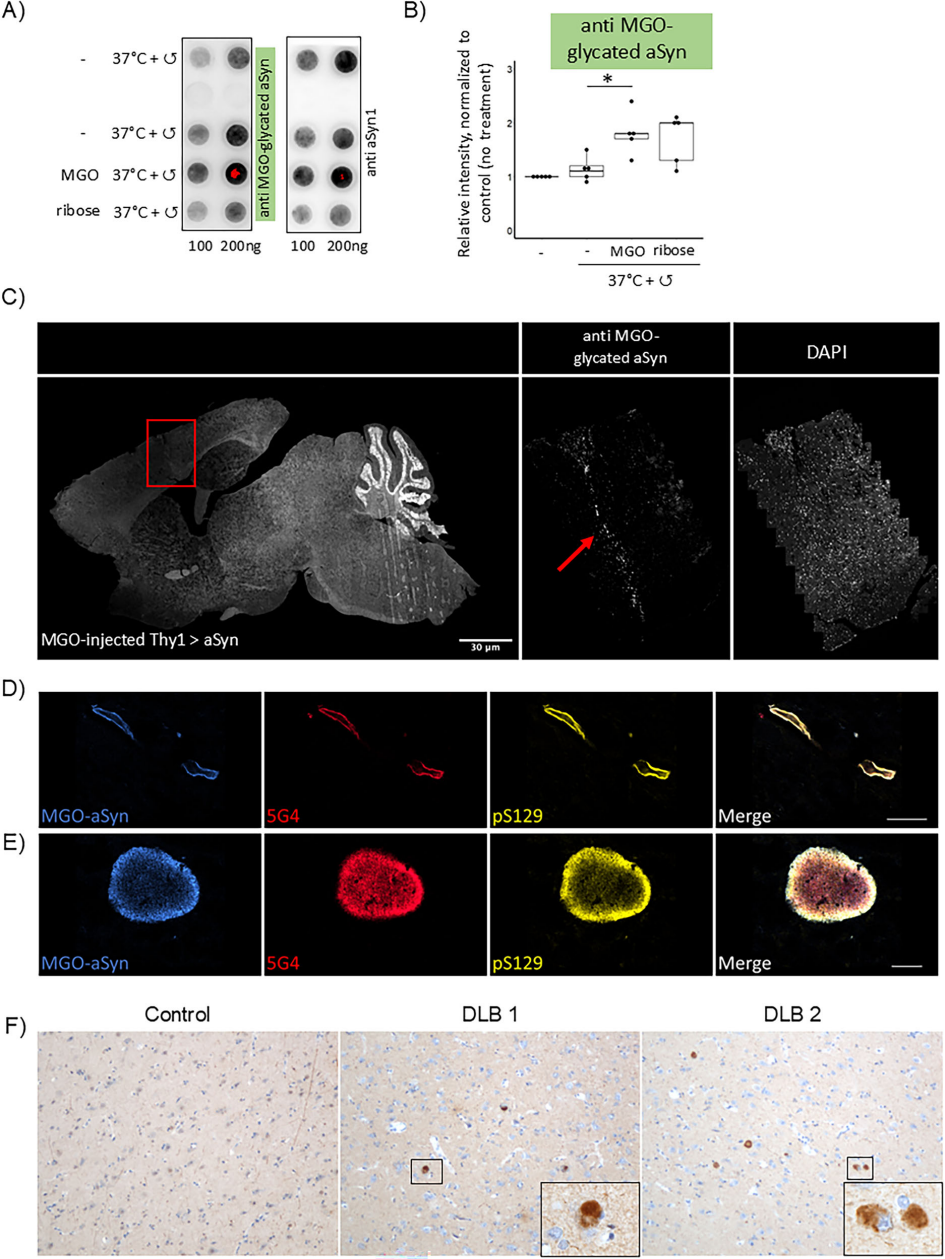
Polyclonal antibody detects glycated aSyn in Lewy neurites and Lewy bodies. **A** Polyclonal rabbit antibody generated against MGO-glycated full-length aSyn (anti MGO-glycated aSyn). **A** Recombinant aSyn was glycated using MGO or ribose. 100 or 200 ng of protein, spotted on a nitrocellulose membrane was stained using the newly produced recombinant antibodies. aSyn1 was used as loading control as shown in the representative blot. **B** Quantification of the relative intensity of all blots, normalized to control condition (*n* = 5, mean ± SD). **C** Transgenic mice, overexpressing aSyn with a Thy1 promoter injected with MGO. Sagittal sections were stained with anti-MGO-glycated aSyn. Anti-MGO-aSyn labeled both Lewy neurites (**D**) and Lewy bodies (**E**) immunoreactive for 5G4 and pS129 and demonstrated a similar pattern of labeling. Scale bars = 30 µm (**C**), 10 µm (**D** and **E**). **F** Detection of aSyn deposits in DLB patients with the Anti-MGO aSyn antibody. Immunohistochemical staining for glycated aSyn in age-matched control and DLB brain samples. Representative sections were stained with anti-MGO-aSyn antibody. All sections were processed under identical conditions. Sections were counterstained in haematoxylin, mounted in DPX and imaged on a Nikon Eclipse 90i microscope. (Scale Bar 200 μm).

Since MGO-modified aSyn consistently exhibited the strongest effects across all models tested, we aimed to develop a polyclonal antibody for detecting glycated aSyn. In our previous work, we demonstrated that MGO glycation primarily affects N-terminal lysine residues in aSyn, specifically K6, K10, K12, K21, K23, K32, K34, K43 and K45^13^. Based on these findings, we attempted to generate a novel polyclonal antibody recognizing MGO-glycated aSyn, which would enable further investigations of its pathological role in disease. To achieve this, rabbits were immunized with an MGO-glycated aSyn, and the resulting antisera were affinity-depleted to increase specificity. The antibody was validated using synthetic peptides of aSyn (peptide 1: residues 5-MKGLSKAKEGVVAAAEKTK-23) and peptide 2: 32- GKTKEGVLYVGSKTKEGVVHGVATVAEKTK-60 and full-length recombinant aSyn, glycated with MGO and ribose (Supplementary Fig. 4A, B, Fig. 5A). The antibody showed binding to MGO-glycated peptides and proteins, while unmodified controls showed little to no signal. The antibody also displayed lower and more variable recognition of ribose-glycated aSyn, supporting its preferential binding to MGO-induced modification, and highlighting differences in the glycation chemistry, and in the species formed (Supplementary Fig. 4A, B, Fig. 5A, B).

To better understand the epitope specificity of the antibody, we tested it on synthetic aSyn peptides 1 and 2 that were synthesized to contain either carboxyethyl lysine (CEL) or carboxymethyl lysine (CML) modifications, two well-characterized glycation end-products^39–41^. These modifications were site-specifically introduced into lysine residues through chemical synthesis, as we previously described^42^.

The antibody robustly recognized CEL-modified peptides (peptides 1-CEL and 2-CEL), but not their CML-modified counterparts (peptides 1-CML and 2-CML), suggesting selective epitope recognition toward MGO-related modifications such as CEL, while excluding structurally distinct glycated forms like CML (Supplementary Fig. 4B).

### MGO-glycated aSyn accumulates in mouse and in human brain tissue

To assess the reactivity of the antibodies in tissue, we used brain tissue from transgenic mice expressing human aSyn under the Thy1 promoter^43,44^ that received intracerebroventricular injections with MGO^20^. Sections from brains of animals injected with vehicle or MGO were analysed blindly. Out of 6 animals that received MGO injections, 5 displayed strong anti-MGO-glycated aSyn immunoreactivity (Supplementary Table 1). Consistently, cells along the needle injection tract, which were more directly exposed to MGO, were strongly stained with anti-MGO-glycated aSyn (Fig. 5C).

Next, we tested the anti-MGO-glycated aSyn antibody in human tissue from the dorsal motor nucleus of the vagal nerve of PD cases, a region amongst the first to be affected by Lewy body pathology in PD^45^. Interestingly, the signal of the anti-MGO-glycated aSyn antibody colocalized with the aSyn antibodies 5G4 (for aggregated aSyn) and pS129 aSyn, labeling Lewy bodies and neurites, the characteristic hallmarks of PD-associated pathology (Fig. 5D, E, Supplementary Table 2).

To further investigate whether glycated aSyn is a feature of disease pathology rather than aging or metabolic stress, we performed immunohistochemistry on brain sections from confirmed cases with dementia with Lewy bodies (DLB) compared with an age-matched control case. Staining with the anti-MGO-aSyn antibody revealed clear detection of aSyn-positive deposits resembling Lewy bodies in both DLB samples. In contrast, no such staining was observed in control brain tissue (Fig. 5F).

Overall, the full-length anti-MGO–aSyn antibody exhibited strong reactivity in both recombinant protein assays and human brain sections from patients with DLB, but showed no detectable signal in age-matched control tissues. The antibody selectively recognized aSyn-positive, Lewy body–like deposits, supporting its potential as a marker for disease-specific glycation events, rather than those associated with normal aging or general metabolic stress.

## Discussion

Here, we investigated molecular effects of glycated aSyn in cell and animal models, and confirmed the presence of glycated aSyn in post-mortem human brain tissue, further supporting an under-studied but likely important pathological role. PD and related synucleinopathies are complex, age-associated neurodegenerative disorders with multifunctional etiology. While environmental and metabolic factors have been implicated in disease risk, the molecular mechanisms linking these contributors to PD pathogenesis remain incompletely understood. Notably, T2DM, and other metabolic conditions characterized by altered sugar homeostasis share common pathological features, including chronic inflammation^46,47^, and are associated with elevated risk for the onset and progression of PD^24,48,49^.

Neuroinflammation is a major contributor to PD onset and progression, involving both innate and adaptive immune responses^50,51^. Anti-inflammatory treatments have shown neuroprotective effects in PD models^52^. The receptor for advanced glycation end products (RAGE), linked to chronic inflammation in neurodegenerative diseases, may also modulate neuroinflammation in PD^53^. Microglia, the brain’s resident immune cells, play a central role by maintaining homeostasis and responding to pathological stimuli. When activated, they release pro-inflammatory cytokines and chemokines, disrupting the blood-brain barrier and affecting neuronal and glial function. Pathological conformational changes and aggregation of aSyn trigger chronic neuroinflammatory responses^54^. In the present study we found that both ribose- and MGO-mediated glycation enhance the impact of aggregated aSyn on microglia activation.

Our study provides new insight into how glycation by MGO or ribose distinctly alters aSyn aggregation and pathogenicity, contributing to PD-relevant phenotypes. Building upon our previous work describing MGO and ribose as potent glycating agents of aSyn, we now showed that MGO-glycated aSyn (aSyn-MGO) and ribose-glycated aSyn (aSyn-ribose) exert markedly different effects on aSyn seeding, aggregation, and downstream cellular responses. Using differentiated SH-SY5Y cells and primary cortical neurons, we observed that aSyn-MGO, but not aSyn-ribose, induces detergent-insoluble HMW species and pS129-positive inclusions. These species partially colocalize with the nucleus, supporting previous findings that MGO-mediated glycation disrupts membrane binding and alters intracellular localization of aSyn. The data further suggest that MGO-glycated aSyn exhibits enhanced seeding activity and pathological potential, in contrast to ribose-glycated aSyn, which remains predominantly soluble and does not induce detectable aggregation in these models. Together, our findings demonstrate that while MGO-glycated aSyn drives aggregation, seeding, and pathological inclusion formation, ribose-glycated aSyn remains largely soluble and only induces mild cytotoxicity, underscoring distinct glycation-dependent mechanisms of aSyn pathogenicity. Both types of glycations induce neuroinflammatory responses, leading to increased expression of pro-inflammatory markers such as IL-6, TNF-α, IL-1β, iNOS, and p62 in microglial cells and affect the MGO-detoxifying system. Interestingly, although aSyn-MGO and monomeric aSyn are taken up by microglia at comparable levels, aSyn-ribose is internalized to a lesser extent, suggesting that the inflammatory activation induced by aSyn-ribose is not paralleled by efficient microglial clearance. One possible explanation is that ribose-mediated glycation alters aSyn’s conformational ensemble and immunogenic profile, thereby reducing recognition by phagocytic receptors such as Toll-like receptors (TLRs) or scavenger receptors B^55,56^. This dissociation between inflammation and clearance is consistent with prior studies showing that certain modified forms of aSyn can trigger immune activation without enhancing degradation^57,58^. Microglia utilize autophagic pathways regulated by TLR4 and NF-κB signaling—to degrade extracellular aSyn^59^. However, PTMs such as glycation may hinder this process by impairing aSyn recognition or altering its intracellular routing.

Our findings underscore the importance of considering how specific biochemical modifications to aSyn affect both its immunogenicity and clearance by microglia. Further studies will be needed to dissect the molecular interactions governing these processes and to explore whether therapeutic modulation of microglial receptors or inflammatory signaling could improve clearance of glycated aSyn species.

Interestingly, both glycated aSyn forms suppressed glyoxalase I (Glo I) enzymatic activity without altering total protein levels, indicating post-translational or functional inhibition of this key detoxifying enzyme. The reduction in Glo I activity observed upon aSyn–MGO and aSyn-ribose treatment may result from direct glycation of Glo I, leading to structural and functional impairment, or indirectly through aSyn–MGO– or aSyn–ribose–induced oxidative stress that perturbs glutathione availability, a cofactor essential for Glo I activity^60–62^. This aligns with previous studies showing Glo I downregulation with aging in human brain^32^, as well as in the substantia nigra of PD patients^33^ and across several PD models^34^. Together, these findings suggest that impaired glyoxalase activity may exacerbate glycation stress, particularly in neurons already burdened by aSyn accumulation. Moreover, aSyn deficiency in mice has been associated with altered levels of advanced glycation end-products (AGEs), increased Glo I expression, and enhanced glycation stress^35^ pointing to the direct involvement of aSyn in sugar metabolism. Consistently, knockdown of aSyn in human iPSC-derived neurons abolished MGO-induced cytotoxicity, further supporting the link between aSyn expression and vulnerability to glycation stress, as previously reported^13^.

The differential effects of MGO and ribose on aSyn seeding and aggregation of aSyn species may be attributable to the distinct AGEs they generate. in cell models. AGEs represent a diverse class of modifications (over 20 structurally unique compounds have been described) that affect protein conformation, aggregation kinetics, and toxicity^63^. Our previous work demonstrated that MGO and ribose affect aSyn aggregation and conformation in different ways resulting in distinct structural and aggregation outcomes^11^. These findings align with studies showing that mutations or other post-translational modifications of aSyn significantly influence its biophysical properties and pathological behavior, causing broad changes in aSyn conformations^64,65^. Glycated aSyn may exert toxic effects through multiple mechanisms, including membrane pore formation, oxidative stress, and resistance to proteasomal degradation, which disrupts proteostasis and promotes autophagy-mediated cell loss. In addition, it activates microglia and engages RAGE/NF-κB signaling, creating a feedback loop that amplifies inflammation and neuronal damage. Together, these effects underscore how glycation contributes to PD pathogenesis, particularly under disease conditions^66^.

To investigate the disease relevance of MGO-glycated aSyn, we developed a novel polyclonal antibody targeting MGO-specific epitopes. The antibody displays strong reactivity toward carboxyethyl lysine (CEL)-modified aSyn but not toward carboxymethyl lysine (CML) modifications, confirming its specificity for MGO-related glycation products. Importantly, this antibody detects Lewy body–like aSyn deposits in DLB patient tissue and in MGO-injected transgenic mouse models, with no signal in age-matched control brains. While this antibody preferentially recognizes glycated aSyn, low-level binding to unmodified aSyn cannot be fully excluded. Thus, our interpretation is that the inclusions observed in PFF-injected mice and human DLB brain likely represent predominantly glycated species. Overall, these findings support the hypothesis that MGO-glycation is associated with disease-specific, rather than aging-related, aSyn pathology. Further characterization of the antibody, including testing against other MGO-modified proteins, will be an important next step to precisely define its specificity.

Our findings also contribute to the growing body of evidence linking T2DM with PD through shared pathophysiological mechanisms such as insulin dysregulation, oxidative stress, mitochondrial dysfunction, and neuroinflammation. Altered insulin signaling has been shown to impair neuronal survival and may be therapeutically targeted in PD^67^. Hyperglycemia promotes mitochondrial ROS production, compromising nigrostriatal neuron viability and suggesting that glycemic control could lower PD risk^68^. Moreover, activation of the NF-κB/NLRP3 inflammasome axis in T2DM drives neuroinflammation and neuronal apoptosis, processes also central to PD^69^. Consistently, our study shows that glycated aSyn, particularly when modified by ribose, can trigger neuroinflammatory responses without robust aggregation, indicating that early-stage glycation may activate inflammatory pathways prior to inclusion formation. These findings highlight that distinct glycation modifications of aSyn may differentially influence its immunogenicity and clearance, providing a mechanistic link between T2DM-associated metabolic fluctuations and PD pathology.

As the connection between diabetes and PD has now been firmly established^24,48,49^, a growing body of literature suggests that, compared to constantly high glucose levels, high fluctuations particularly are more deleterious^70–73^. While swings of glucose levels are a regular physiological phenomenon, glycemic variability is increased in prediabetic and diabetic individuals^74^. Furthermore, even individuals not classified as diabetic or prediabetic experience peaks of glucose levels in the prediabetic and diabetic range^75,76^. Given that high glucose levels lead to increased MGO levels, it is reasonable to assume that high glycemic variability also causes increased MGO-mediated glycation^77–79^. But how can this be measured in cell and animal models? Measuring MGO levels is possible but difficult, and the consequences of aSyn glycation have been described^80^. However, directly measuring the glycation status of aSyn has been challenging. Many studies have used antibodies generated against glycated lysine residues in BSA^20,81–83^, but they are not aSyn-specific. Other antibodies were developed to specifically detect certain aSyn conformations^84–86^. However, antibodies directed against glycated aSyn have not been previously described, and could be important tools for studying the interplay between diabetes and PD at the molecular level. Our study addresses this limitation by introducing a novel polyclonal antibody raised against full-length MGO-glycated aSyn, capable of detecting pathological inclusions in human DLB brain tissue. This novel tool enables the specific identification of glycated aSyn species in situ, an essential step in dissecting their role in PD pathogenesis. This is particularly relevant given the interest in testing glucagon-like peptide-1 (GLP-1) receptor agonists, such as lixisenatide, in people with early PD^87^. This illustrates that the connection between diabetes and PD is not only crucial for understanding the onset of PD but also that understanding of the molecular interactions can aid in treatment strategies. However, despite our promising findings, the human tissue data are currently limited to a small number of samples. Validation in larger patient cohorts will be necessary to establish the diagnostic utility of the anti–MGO-aSyn antibody and to more fully define the role of glycation in PD pathogenesis. While our study focused on characterizing the effects of glycated aSyn in cellular and ex vivo models, future work could extend these findings by examining diabetic-like metabolic conditions, evaluating anti-glycation interventions, and confirming MGO delivery and effects in vivo. These studies should aim to clarify the mechanisms underlying selective glycation patterns, their impact on protein conformation and toxicity, and their interaction with cellular clearance and immune pathways. Such efforts may pave the way for the development of novel biomarkers and for precision therapies aimed at mitigating glycation stress in PD and related disorders.

## Methods

### Production of polyclonal antibodies

A polyclonal antibody specific for MGO-glycated alpha-synuclein (aSyn-MGO) was generated in collaboration with Davids Biotechnology (Regensburg, Germany). Full-length recombinant aSyn was glycated with methylglyoxal as we previously described^13^, and used as the immunogen for rabbit immunization following established protocols. The resulting antibody was affinity-purified against MGO-glycated full-length aSyn to ensure specificity.

### Generation of synthetic peptides containing CEL or CML

Generation of CEL- and CML modified peptides is described in ref. ^42^. In brief, solid-phase peptide synthesis was combined with orthogonal protection of amino acid side-chain functionalities and reductive amination strategies to generate 4 different peptides: Peptides 1 and 2 with CEL- or CML-modified lysines, respectively.

### Production and purification of aSyn

Production and purification of recombinant aSyn was performed as described^88^. In brief, pET21-aSyn was transformed into competent *E. coli* BL21-DE3 (Sigma), *E. coli* was grown in 2x LB medium with ampicillin (200 μg/mL) at 37 °C with constant shaking. At an OD_600_ of 0.5–0.6, the expression was induced with 1 mM of isopropylβ-thiogalactopyranoside. After 2 h, bacteria were pelleted by centrifugation at 6600 × *g*, 15 min and lysed on ice in 10 mM Tris pH7.6, 750 mM NaCl, 1 mM EDTA with protease inhibitor (cOmplete, Roche). Lysates were sonicated on ice for a total of 5 min (30 s on, 30 s off pulses, 60% power), and heated for 15 min at 95 °C. Samples were then centrifuged at 15.000 g and the supernatant was subjected to dialysis in 10 mM TRIS pH 7.6, 1 mM EDTA, 50 mM NaCl. Anion exchange chromatography (HiTrap Q HP, Cytiva) was performed with a mobile phase of 25 mM Tris pH 7.6 and a linear gradient of 9 column volumes of elution buffer to 1 M NaCl on an Äkta Pure 25 M (Cytiva). Fractions containing pure aSyn were identified on a Coomassie-stained SDS-PAGE and pooled. Proteins were further purified with size exclusion chromatography using a HiLoad Superdex200pg column (Cytiva). Fractions containing pure aSyn were identified on a Coomassie-stained SDS-PAGE and pooled and extensively dialyzed into water. Following lyophilization (Zirbus), protein was stored at −20 °C. aSyn concentration was measured using absorbance at 280 nm (molar extinction coefficient 5960 M^–1^ cm^–1^).

### Glycation of recombinant aSyn

100 µM aSyn was dissolved in 1xPBS, 7.4 with 3.7 mM EDTA with or without 5 mM MGO (Sigma) or 0.8 M ribose and filtered with 0.2 μM membrane syringe filter. The samples were then incubated for five days at 37 °C, under constant agitation (300 rpm) in low binding tubes (Corning Incorporated). Glycation was confirmed via measuring fluorescence as described in ref. ^11^. Briefly, Fluorescence of glycation products was measured by excitation at 340 nm and emission at 390 nm. The reported concentrations of glycated aSyn were calculated based on the initial amount of monomeric protein used prior to glycation, under the assumption that no material was lost during incubation.

### Cell cultures

The human neuroblastoma (SH-SY5Y) cells used conditionally expressed human wildtype aSyn under a Tet-Off cassette^27^. Cells were grown in RPMI with 1 μM doxycycline, 10% FBS, and 1% penicillin/streptomycin at 37 °C, 5% CO_2_. To induce aSyn expression from the Tet-Off cassette, doxycycline was omitted. For differentiation, cells were seeded in 6-well plates. One day after plating, the medium was supplemented with 10 μM retinoic acid in RPMI supplemented with 0.5% FBS and 1% penicillin/streptomycin, at 37 °C, 5% CO_2_ for 4 days. Media was changed every second day. Following differentiation, cells were treated with 100 nM of the respective glycated aSyn species for 4 days. aSyn treated the same way but without glycating agents was used as control. Mild trypsinization for 5 min was used to remove excess unbound aSyn protein. Cultures were washed with PBS and fixed with 4% paraformaldehyde (PFA) for 20 min at RT.

### Primary rat neuronal cultures

In total, 7 to 12 primary cortical neurons were prepared from embryonic day E18 Wistar rat brains. After dissection in Hank’s Balanced Salt Solution (HBSS; Gibco), the cortices were digested by Trypsin (Gibco) for 15 min at 37 °C, followed by addition of 100 μl DNase I (10 mg/ml; Roche) and 100 μl FBS (Life Technologies), mixed by inverting and spun down. The liquid was replaced with 1 ml FBS and the tissue triturated with a pasteur pipette. The dissociated cells were spun down and transferred into fresh culture medium (Neurobasal medium, Gibco), with 2% B27 supplement (Gibco), 0.5mM L-glutamine (200Mm Gibco), and 1% penicillin/streptomycin (Gibco). Cells were plated in in 24- or 6-well plates previously coated with Poly-L-ornithin (0,1 mg/ml, Sigma-Aldrich) at a density of 250,000 cells/mL. The cultures were differentiated for 5 days. At day 5, aSyn species were added to a final concentration of 50 nM and incubated for 20 days. Mild trypsinization for 5 min was used to remove excess unbound aSyn material. Cultures were washed with PBS and fixed with 4% paraformaldehyde (PFA) for 20 min at RT.

### Primary microglial cultures

Primary microglia were obtained from mixed glial cell cultures from C57BL6 wild-type newborn mice as described previously^89^. Briefly, brains were isolated in 1xHBSS (Gibco Invitrogen), meninges were removed, and the brains were washed three times with HBSS (without Ca^2+^, Mg^2+^, and phenol; PAN Biotech). The tissue was trypsinized (0.05% trypsin-EDTA; PAN Biotech) at 37 °C for 10 min. After aspirating the trypsin solution, the digestion was stopped by adding 0.5 mg/mL DNase I (Roche) in microglia medium (DMEM (PAN Biotech), supplemented with 0.5% penicillin-streptomycin (PAN Biotech) and 10% FBS (Anprotec)). Tissue was incubated for three additional minutes at 37 °C and homogenized with a glass pipette. Cells were spun down at 800 g for 10 min, the pellet was resuspended in medium. Cells were plated into T75 flasks (Corning, Merck) that had previously been coated with poly-L-ornithine (Sigma-Aldrich, 0.1 mg/mL in borate buffer) overnight at 37 °C and 5% CO_2_. The following day (DIV2), the cells were washed three times with pre-warmed HBSS solution (PAN Biotech) and once with microglia medium before new medium was added. At DIV3, cell medium was replaced once more. At DIV5, the culture was stimulated by substituting one third of the medium with L929 medium (L929 mouse fibroblast cells had previously been plated in T175 cm^2^ cell culture flasks (Corning, Merck) with 100 mL culture medium (DMEM, PAN biotech; supplemented with 10% FBS (Anprotec) and 1% penicillin-streptomycin (PAN Biotech)) for seven days at 37 °C with 5% CO_2_. The media was then collected, sterilized by filtration using a 0.22 μm filter (Sartorius, Göttingen, Germany) and stored at –20 °C). At DIV8, microglia were harvested by mild shaking and collected (10 min at 800 g) and replated into culture plates previously coated with poly-L-ornithine (Sigma-Aldrich, 0.1 mg/mL in borate buffer). Microglia were allowed to attach to the new flasks for 16 h and treated with 100 nM aSyn species for 24 h.

### RNA isolation and qRT-PCR

RNA was extracted from microglial cultures using TRIzol Reagent according to the manufacturer’s instructions (Invitrogen). cDNA was reverse transcribed using QuantiTect Reverse Transcription kit (Qiagen, MD, USA) according to the manufacturer’s instructions. qPRC was performed on an Applied Biosystems Real-Time PCR Systems using SYBR Green Master Mix (Qiagen); 95 °C for 10 min, then 40 cycles at 95 °C for 15 s and 60 °C for 25 s. The following primers were used: IL-6 F: ATCCA GTTGC CTTCT TGGGA CTGA, IL-6 R: TAAGC CTCCG ACTTG TGAAG TGGT, IL-1β F: TCATT GTGGC TGTGG AGAAG, IL-1β R: AGGCC ACAGG TATTT TGTCG, TNFα F: CCCTC TCATC AGTTC TATGG, TNFα R: GGAGT AGACA AGGTA CAACC, *β*-actin F: GCGAG AAGAT GACCC AGATC, and *β*-actin R: CCAGT GGTAC GGCCA GAGG. *β*-actin was used as reference gene to calculate the fold change in expression levels using the 2–ΔΔCT method.

### Dot blots

For the dot blots, the protein/peptide specified was diluted in 100 µL PBS and spotted onto a nitrocellulose membrane, (0.1 µm for peptides, 0.2 µm for proteins; Amersham Protran) using a custom-made dot blot apparatus with vacuum. The membranes were completely air-dried and incubated at room temperature with blocking solution. If peptides were used, performing aSyn staining as a loading control was not possible. In this case, the Pierce™ Reversible Protein Stain Kit was used according to manufacturer’s instructions.

Primary antibodies were incubated over night at 4 °C with mild agitation. The polyclonal antibodies generated were used in a concentration of 1 µg/mL and diluted in blocking buffer (0.1 M sodium acetate, 5% BSA, 0.05% Tween-20). Syn1 (BD Transduction Laboratories, 1:1000) was used to assess total aSyn concentration. TBS-Tween (1 × TBS, supplemented with 0.05% (v/v) Tween-20) was used for three 15-min washes. Secondary antibodies (anti-mouse and anti-rabbit IgG, 1∶10,000 in blocking buffer) were applied for one hour at room temperature. After three more washes in TBS-Tween, membranes were developed using Fusion Fx (Vilber Lourmat) with Immobilon Western Chemiluminescent HRP Substrate (Merck Millipore).

### Sequential protein extraction

Cell pellets were collected and solubilized in 1% Triton X-buffer (50 mM tris pH 7.6, 150 mM NaCl, 2 mM EDTA, 1% Triton X-100, protease- and phosphatase inhibitors), followed by a 30-min incubation on ice. Cell lysates were spun down at 13,000 × *g* for 30 min, 4 °C. The supernatant is the Triton X soluble fraction. The pellet was washed with ice cold PBS, resuspended in 2% SDS buffer (50 mM tris pH 7.6, 150 mM NaCl, 2 mM EDTA, 2% SDS, protease- and phosphatase inhibitors), sonicated and incubated at room temperature for 30 min. After centrifugation at 13,000 × *g* for 10 min, the supernatant - the SDS soluble fraction—was collected. Bradford protein assay was used to determine the protein concentration and the samples were then subjected to Western blot analysis.

### SDS-PAGE and immunoblotting

For total protein cell lysates, cells were washed with PBS and lysed on ice in radio-immunoprecipitation assay buffer (RIPA) (50 mM Tris pH 8.0, 150 mM NaCl, 0.1% Sodium-Dodecyl-Sulfate (SDS), 1% Nonidet P40, 0.5% Sodium-Deoxycholate, protease inhibitors, Roche Diagnostics, Mannheim, Germany). Lysates were centrifuged at 10,000 rpm and 4 °C for 10 min and post-nuclear supernatants were kept. Protein concentration was determined using the Bradford assay (BioRad). All samples were measured in triplicate. Equal protein amounts of denatured samples (5 min at 95 °C in 5x protein sample buffer; 125 mM of 1 M Tris HCl pH 6.8, 4% SDS 0,5% Bromophenol blue, 4 mM EDTA 20% Glycerol 10% β-Mercaptoethanol) were subjected to SDS-PAGE on 12% separating gels with 7% stacking gels, using Tris-Glycine SDS 0.5% running buffer (250 mM Tris, 200 mM Glycine, 1% SDS, pH 8.3). The transfer was carried out to 0.45 μm nitrocellulose membranes for 20 min per membrane at constant 25 mA in a semi-dry transfer chamber Trans-Blot® Turbo™ Transfer Solution from Bio-Rad (Bio-Rad Laboratories, Inc., Hercules, CA, USA). Membranes were blocked in 5% (w/v) skim milk (Fluka, Sigma-Aldrich, St. Louis, MO, USA) dissolved in 1xTBS-Tween (50 mM Tris (hydroxymethyl)-aminomethane (TRIS) supplemented with 0.05% (v/v) Tween-20) for 1 h at RT. Incubation with the primary antibodies (anti-aSyn Syn-1 mouse, BD Transduction Laboratories 1:1000; anti-pS129-asyn Rabbit Abcam Ab51253; mouse anti-*β*-actin, 1:10.000, Sigma Aldrich) was performed overnight at 4 °C in 5% Albumin Bovine Fraction V (BSA)/TBS-Tween. Secondary antibodies (anti-mouse and anti-rabbit IgG, 1∶10000 in TBS-Tween) were applied after three times washing in TBS-Tween, for 1 h at RT. Membranes were visualized using Fusion Fx (Vilber Lourmat, Marne-la-Vallée, France) with Immobilon Western Chemiluminescent HRP Substrate (Merck Millipore, Billerica, MA, USA). Protein levels were quantified using ImageJ and normalized to the *β*-actin levels.

### Immunofluorescence analyses

Sagittal sections (30 mm) from transgenic (Thy1-aSyn) mice that had received an intracerebroventricular MGO injection were prepared and processed as described previously^20^. After blocking (0.1 M sodium acetate, 5% BSA, 0.05% Tween-20) at room temperature for one-hour, primary antibodies (used in a concentration of 1 µg/mL and diluted in blocking buffer) were applied overnight. After three 10 min washes with PBS, the sections were incubated with secondary antibodies (diluted 1:2000 in blocking buffer) for 1 h at room temperature. Finally, the sections were washed again three times for 10 min, stained with DAPI for 5 min and cover slipped with Mowiol. For immunofluorescence of cell cultures, cells were grown on glass coverslips, washed and fixed as described above. Permeabilization was performed with 0.5% tritonX 100 at room temperature for 20 min. Coverslips were blocked with 1.5% bovine serum albumin for two hours. Primary antibodies (Tuj1—1:3000, Covance; pS129—1:2000, Abcam51253; Syn1—1:2000, Syn1—1:2000, BD Transduction Laboratories) diluted in 1.5% BSA were incubated overnight at 4 °C. The coverslips were washed three times with PBS and incubated for two hours with secondary antibodies: anti-mouse and rabbit Alexa Fluor 568- and Alexa Fluor 488- conjugated (1:2000, Life Technologies-Invitrogen). Finally, cells were stained with DAPI for 5 min and cover slipped with Mowiol. Images were analyzed using LAS AF v.2.2.1 (Leica Microsystems) software.

### Immunostaining of human brain samples

Human *post-mortem* brain tissue was obtained from Newcastle Brain Tissue Resource, a UK Human Tissue Authority-approved brain tissue repository. Formalin-fixed paraffin-embedded tissue was obtained from the medulla oblongata of two PD cases and cut at 5 µm sections for immunofluorescent analysis. Sections were dewaxed in Histoclear (National Diagnostics, NC, USA) and rehydrated through graded ethanol solutions until water. Antigen retrieval was performed by boiling sections in citrate buffer pH6 for ten minutes prior to immersion in formic acid for five minutes. Sections were blocked in 10% normal goat serum in TBS-T for one hour at room temperature and then incubated in the following primary antibodies suspended in blocking buffer overnight at 4 °C: pS129 (El-Agnaf laboratory, 1 µg/ml^90^) and 5G4 (MABN389, Merck, 2 µg/ml) combined with either anti-MGO-aSyn (1 µg/ml). Primary antibodies were washed off in TBS-T and sections were then incubated in the following secondary antibodies diluted 1:100 in blocking buffer for one hour at room temperature: (goat anti-rabbit-AF405, A48254, Thermo; goat anti-mouse IgG1-AF647, A21240, Thermo; goat anti-mouse IgG2a-AF546, A21133, Thermo). Following secondary antibody exposure, sections were washed and lipofuscin autofluorescence blocked with 0.3% Sudan Black B solution. Sections were visualized on a Leica SP8DLS confocal microscope.

### Immunohistochemistry for detection of glycated aSyn in human brain tissue

Immunohistochemistry was performed on formalin-fixed, paraffin-embedded brain sections from clinically diagnosed DLB patients and age-matched controls to detect glycated aSyn. Polyclonal anti-MGO-aSyn used at the dilution of 1:20. Signal detection was carried out using MACH4 Universal HRP-polymer kits (CellPath, Powys, UK; cat. PBC-BRR534AG) and Betazoid DAB Chromogen kits (CellPath, Powys, UK; cat. PBC-BDB2004MM) according to the manufacturer’s protocols. Sections were counterstained with hematoxylin, mounted in DPX, and imaged using a Nikon Eclipse 90i microscope.

### Determination of glyoxalase I activity

The quantitative determination of Glyoxalase I (Glo I) activity in SH-SY5Y and rat primary neuronal cultures was performed using a Glyoxalase I Activity Assay Kit (Colorimetric) (K591-100, BioVision, San Diego, CA, USA) according to the manufacturer’s protocol.

### LDH cytotoxicity assay

Cells were incubated under the different conditions and cytotoxicity assessed by measuring the cell supernatant with LDH cytotoxity detection kit (Roche Applied Science) according to the manufacturer’s protocol. The samples were measured in triplicates on an Infinite M200 fluorescence plate reader, TECAN plate reader.

### Statistical analysis

Data were obtained from at least three independent experiments and are shown as mean values ± standard deviation (SD). Two-group comparisons were performed using Student’s *t* test, multiple-group comparisons were performed using ANOVA with post hoc Tukey. *p* < 0.05 was considered statistically significant (* *p* < 0.05; ** *p* < 0.001; *** *p* < 0.0001). Statistical analyses were performed in Excel and R.

## Supplementary information

Supplementary Information

## Data Availability

The data obtained in this research are available from the corresponding author upon reasonable request.
