# Supplementary material for: Glycation of alpha-synuclein enhances aggregation and neuroinflammatory responses
Source: NPJ Parkinsons Dis. 2025 Oct 23;11:307. doi: 10.1038/s41531-025-01159-w (PMC12550043; doi:10.1038/s41531-025-01159-w)
Supplement: Supplementary file 1 — Supplementary Information [file 41531_2025_1159_MOESM1_ESM.pdf]

Glycation of alpha-synuclein enhances aggregation and neuroinflammatory responses

Eftychia Vasili[1], Annekatrin König[1], Mohammed Al-Azzani[1], Clara Bosbach[1,2], Luisa Maria Gatzemeier[1,2], Searlait Thom[4], Ana Chegão[3], Hugo Vicente Miranda[3], Claudia Steinem[2], Daniel Erskine[4], and Tiago F. Outeiro[1,4,5,6"]

1. University Medical Center Gottingen, Department of Experimental Neurodegeneration, Center for Biostructural Imaging of Neurodegeneration, Göttingen, Germany.
2. Institute of Organic and Biomolecular Chemistry, Georg-August-University Göttingen, Tammannstraße 2, 37077 Göttingen, Germany.
3. iNOVA4Health, NOVA Medical School, NMS, Universidade NOVA de Lisboa, Lisboa, Portugal
4. Translational and Clinical Research Institute, Newcastle University, Newcastle upon Tyne NE1 7RU, United Kingdom
5. Max Planck Institute for Multidisciplinary Sciences, Göttingen, Germany.
6. Scientific employee with an honorary contract at Deutsches Zentrum für Neurodegenerative Erkrankungen (DZNE), Göttingen, Germany.

Running title

aSyn glycation in Parkinson`s disease

Keywords

Parkinson’s disease, alpha-synuclein, neurodegeneration, glycation, protein aggregation, diabetes

Supplemental information - Figures titles and Legends

Supplementary Figure 1

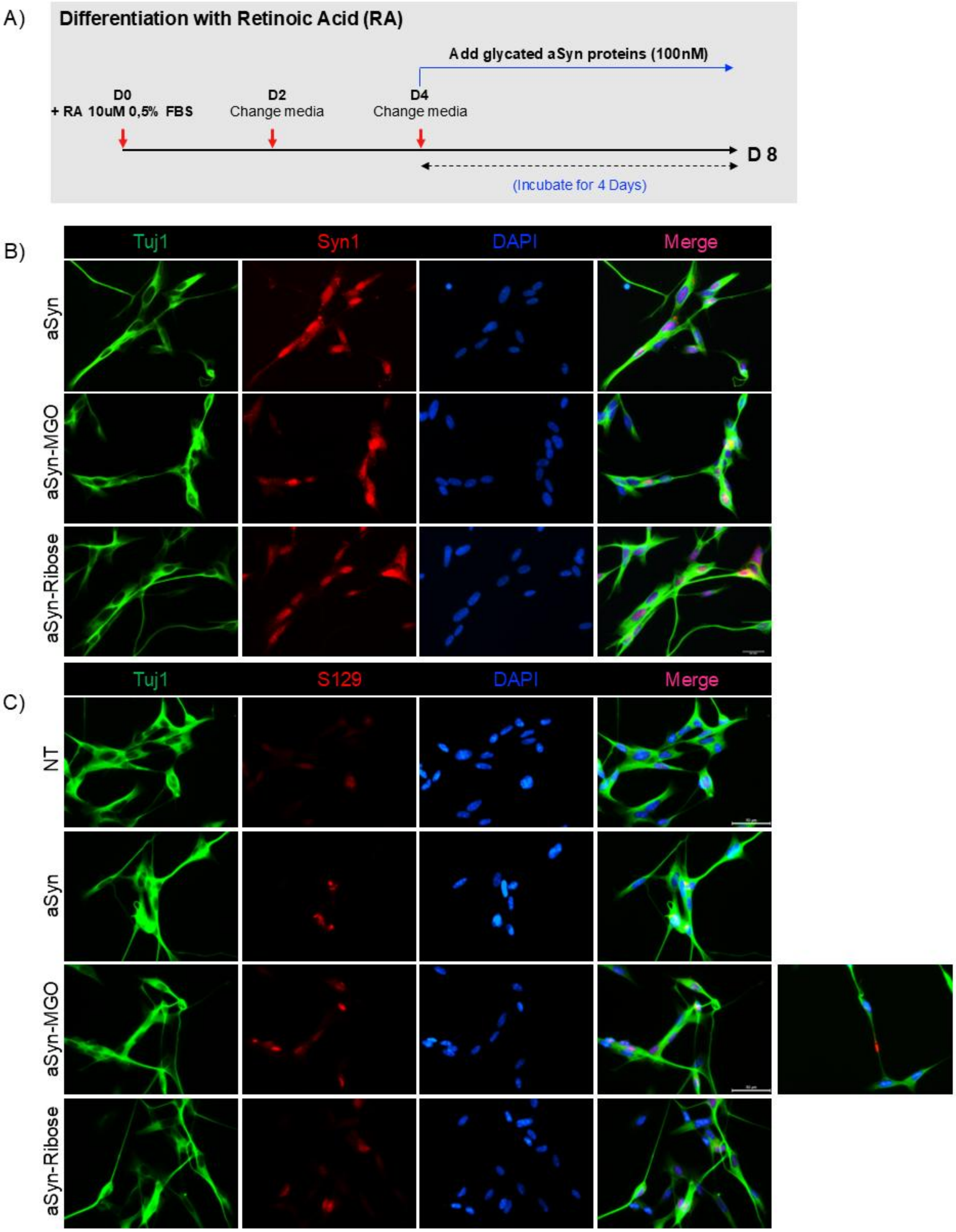

Supplementary Figure 1. SH-SY5Y cells, conditionally expressing aSyn can be differentiated to neuron-like cells A). Schematic illustration of the followed differentiation protocol of the SH-SY5Y cells (doxycycline was omitted from the medium). SH-SY5Y cells, conditionally expressing aSyn were differentiated to neuron-like cells, expressing human wildtype aSyn. aSyn, that had been incubated with or without glycating agent was added to the cells at a final concentration of 100nM for 4 days. B). C). Cells were immunostained using Tuj1 and Syn1 (B) or Tuj1 and pS129 (C). Representative images from n=3 (Scale bar 50μm).

Supplementary Figure 2

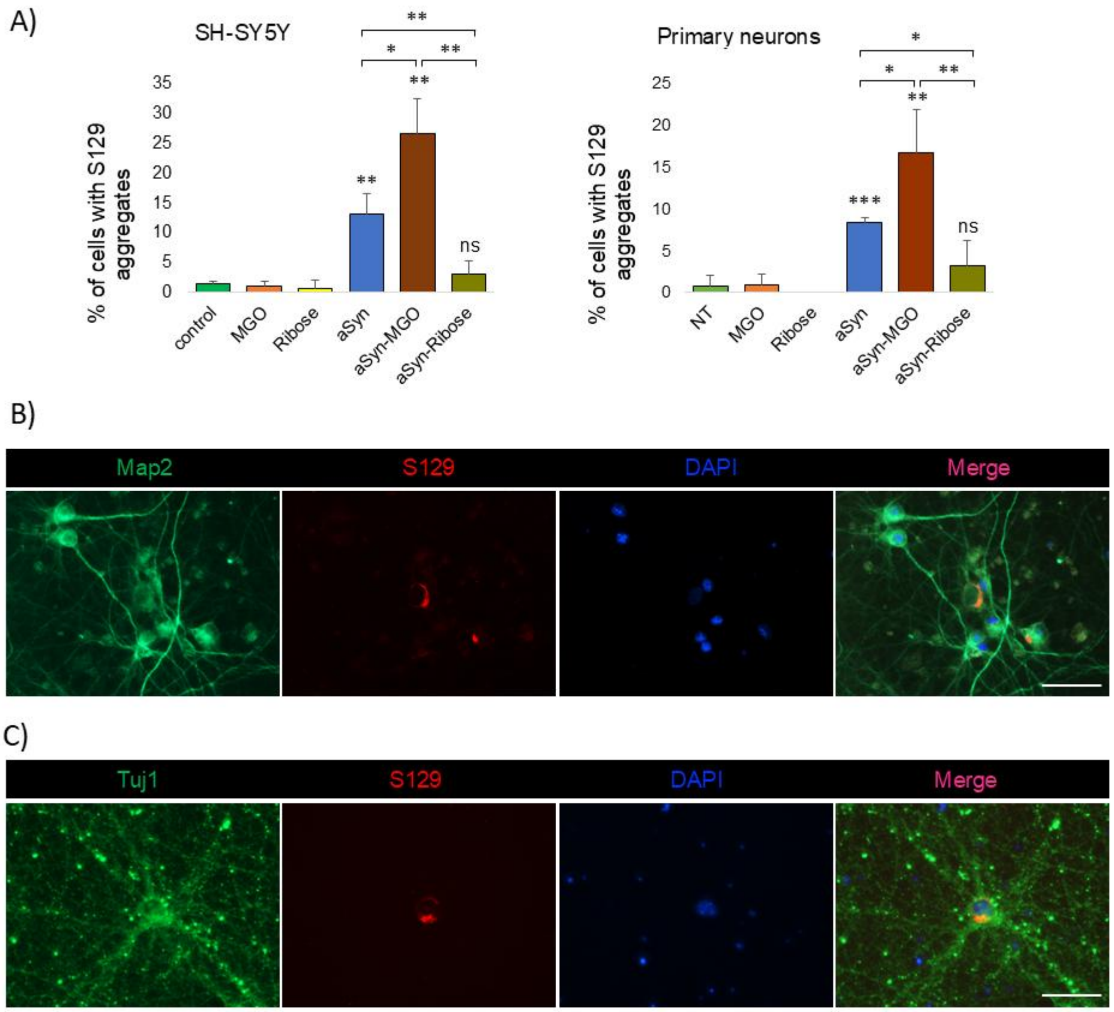

Supplementary Figure 2. Primary rat cortical neurons incubated for 20 days with aSyn pre-formed fibrils (PFFs, 50nM). A). Quantification of S129 positive aggregates in SH-SY5Y cells and primary neurons formed under treatment with glycated aSyn (n = 3, mean ± SD). B). Representative images from cells immunostained with antibodies against Map2 and pS129 aSyn (C) or Tuj1 and pS129 aSyn. (Scale bar 50μm).

Supplementary Figure 3

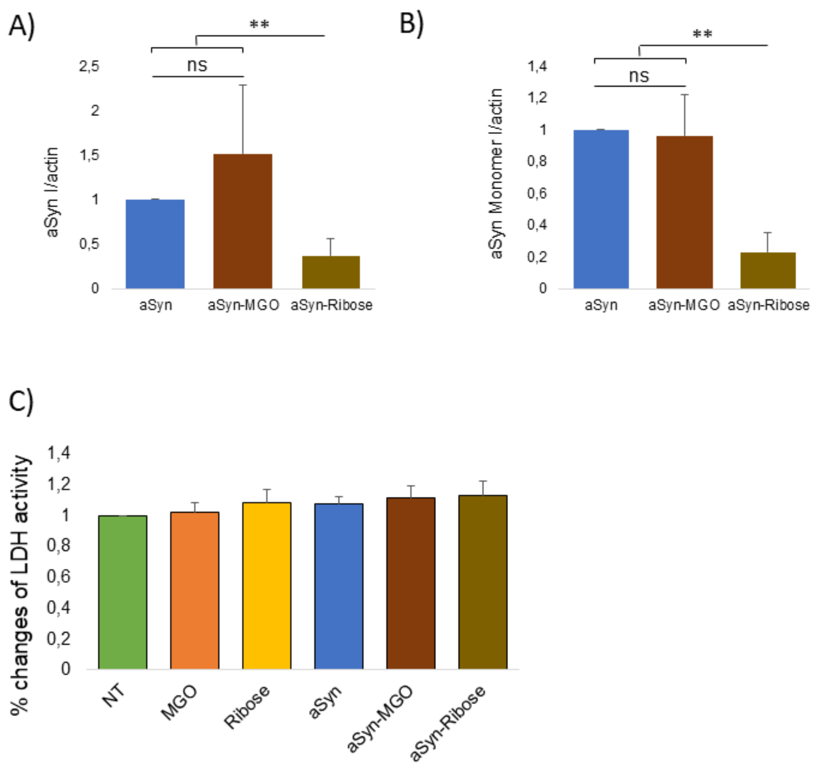

Supplementary Figure 3. Microglia were treated with 100nM aSyn species for 24 hours. RIPA protein extracts of microglia extracts were examined with immunoblotting for Syn1. β-actin is used as a loading control and the intensity of Syn1 signal (A. full lane B. Syn1 monomer only) was quantified (n=4, mean ± SD). C) Cytotoxicity under different conditions of treatment was measured by the activity of released LDH and is displayd as per cent cytoxicity (n=6).

Supplementary Figure 4

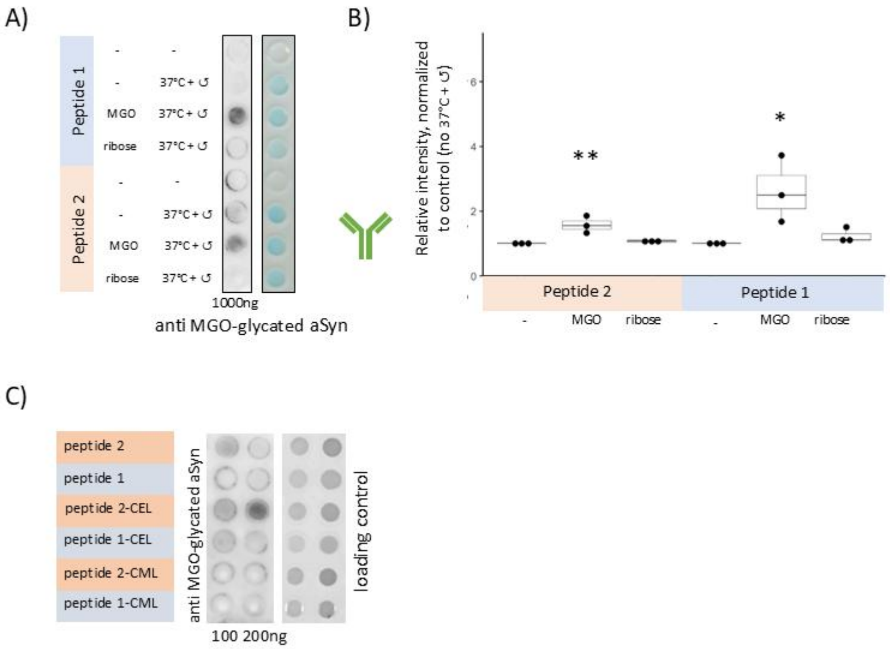

Supplementary Figure 4. Synthetic peptides, glycated with MGO or ribose are detected by the polyclonal anti MGO-glycated aSyn antibody generated. The antibody anti MGO-glycated aSyn was used to stain MGO or ribose glycated peptides. The peptides are depicted in blue (peptide 1) and red (peptide 2). The glycation protocol involves a 5-day incubation with mild agitation at 37°C. Signals were normalized to peptides that had been incubated without glycating agent (A and B). Since the peptides cannot be detected with most aSyn specific antibodies, a highly sensitive commercial staining kit was used as loading control. Mean ± StDev is displayed, n=3. C. Synthetic AGEs are recognized by the polyclonal antibody. Synthetic peptides containing CEL-lysine or CML-lysine residues

(abbreviated as peptide 1-CEL/CML or peptide 2-CEL/CML) were spotted on nitrocellulose membranes. Anti-MGO-glycated aSyn used for staining. The Pierce™ Reversible Protein Stain Kit was used as loading control (n=3).

Supplementary Table 1.

	Vehicle			MGO		
**WT**	P5	P2	P4	A5	A2	A4
**Thy1 aSyn**	V7	V8		E9	E5	E6

Supplementary Table 1. The novel polyclonal antibody generated to detect MGO-glycated aSyn in mouse brain sections. Transgenic animals and control littermates received intracerebroventricular injections of MGO or vehicle[1]. Sagittal sections of 12 animals were stained with anti-MGO-glycated aSyn and analysed blindly. Blue shading indicates anti MGO-glycated aSyn staining with staining along the ventricle and along the injection channel. Importantly, animal V10 was classified as an outlier due to unusually-low cell numbers, and was excluded.

Supplementary Table 2.

Case	Age	Sex	PM Delay (hrs)	Braak Tangle Stage	Thal Phase	Braak LB Stage	Cause of Death
Control	92	F	50	3	5	0	Respiratory infection
DLB 1	74	M	24	2	3	6	Complications of DLB
DLB 2	73	M	47	3	1	6	Bronchopneumonia

Supplementary Table 2. Clinical and neuropathological information for human brain samples used in the study. Demographic, postmortem, and neuropathological characteristics of human brain samples used in this study. PM delay: postmortem delay in hours; Braak tangle and LB stages indicate neurofibrillary tangle and Lewy body pathology, respectively; Thal phase refers to amyloid-β plaque distribution.

Supplementary Figure 5 – Part I

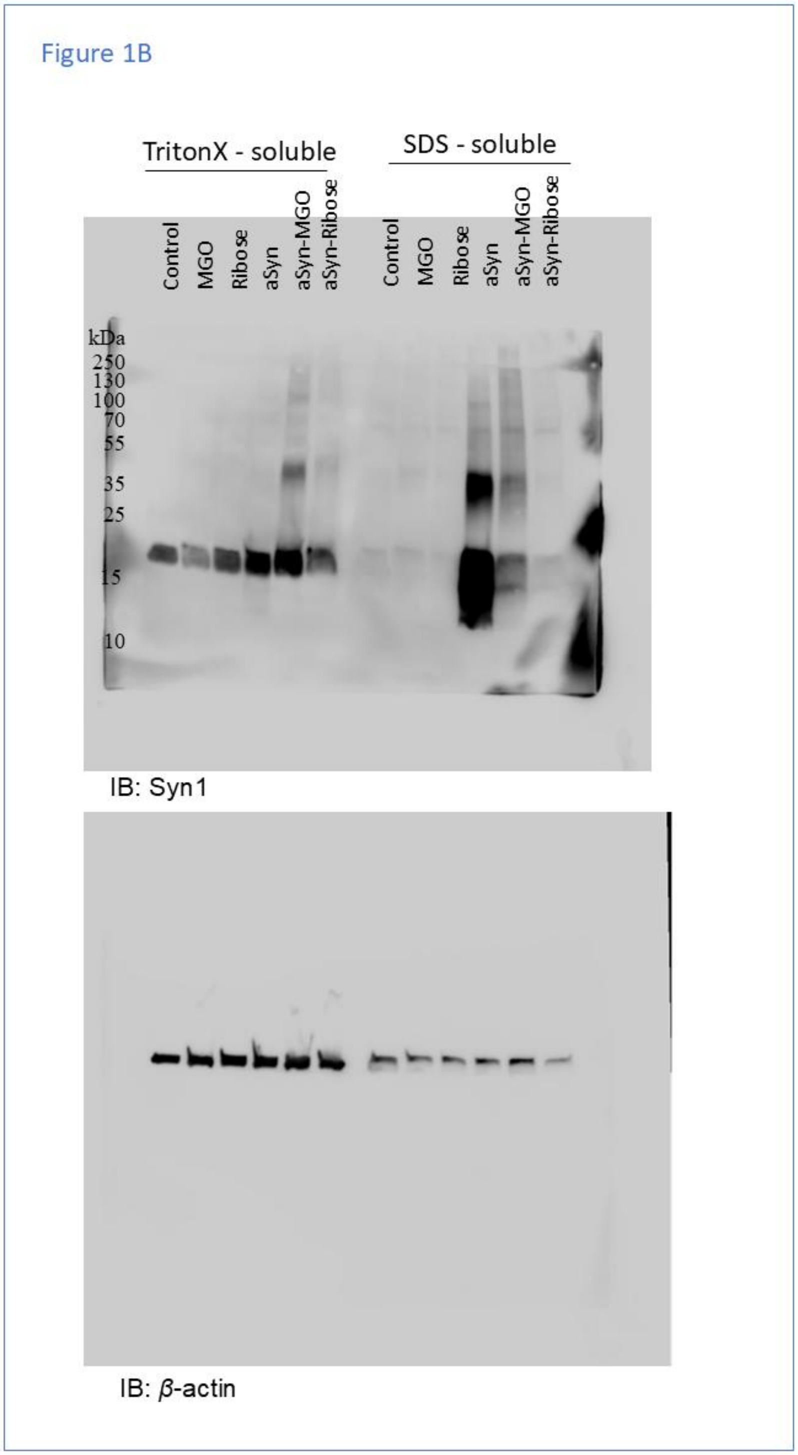

Supplementary Figure 5 – Part II

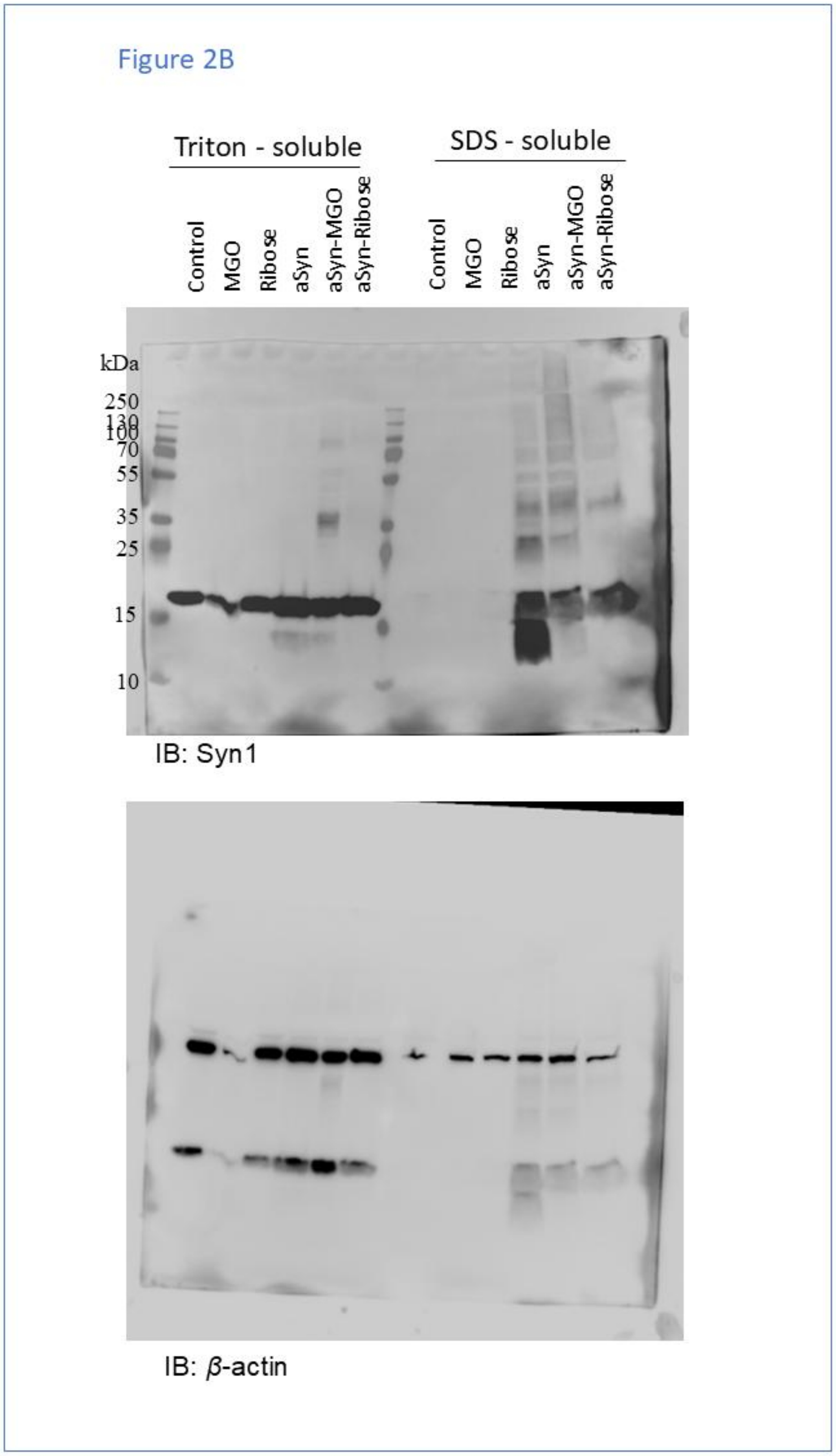

Supplementary Figure 5 – Part III

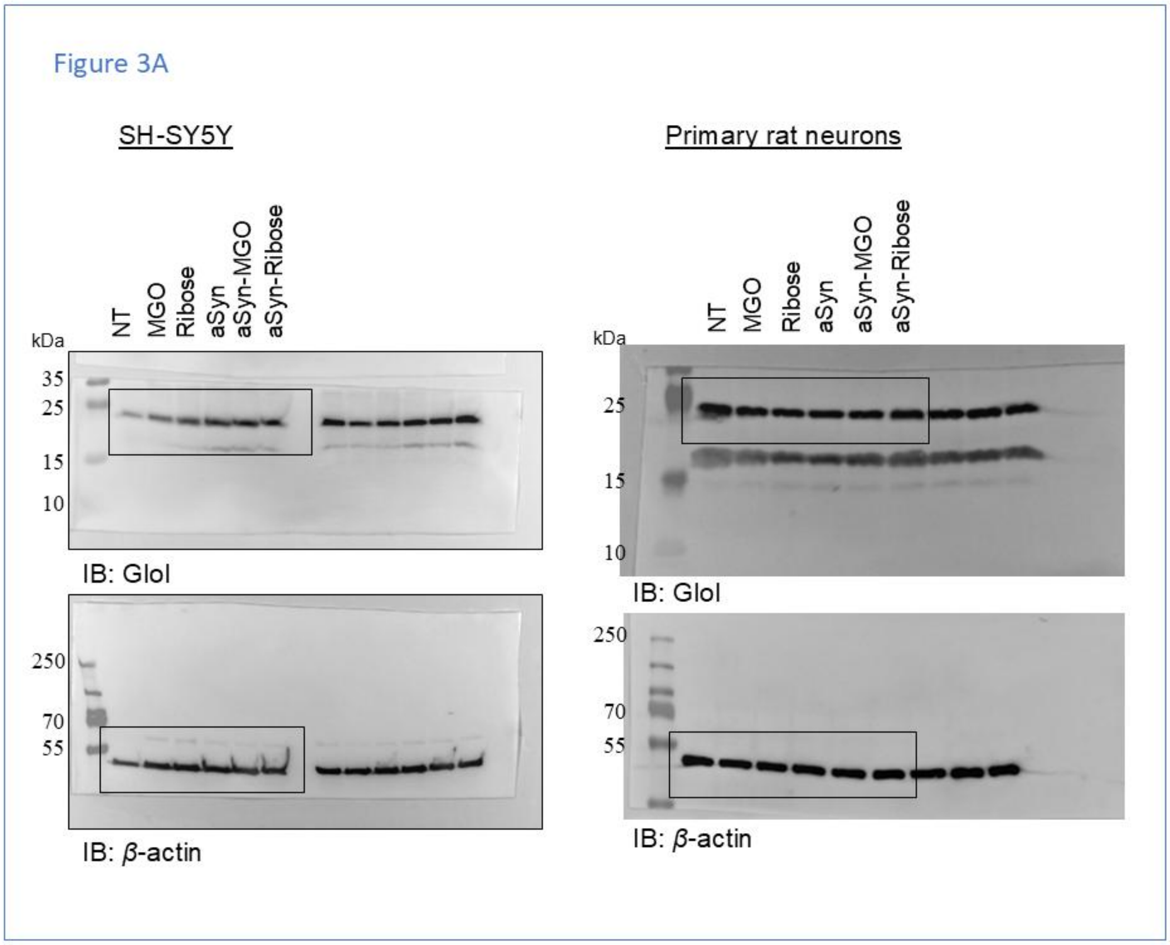

Supplementary Figure 5 – Part IV

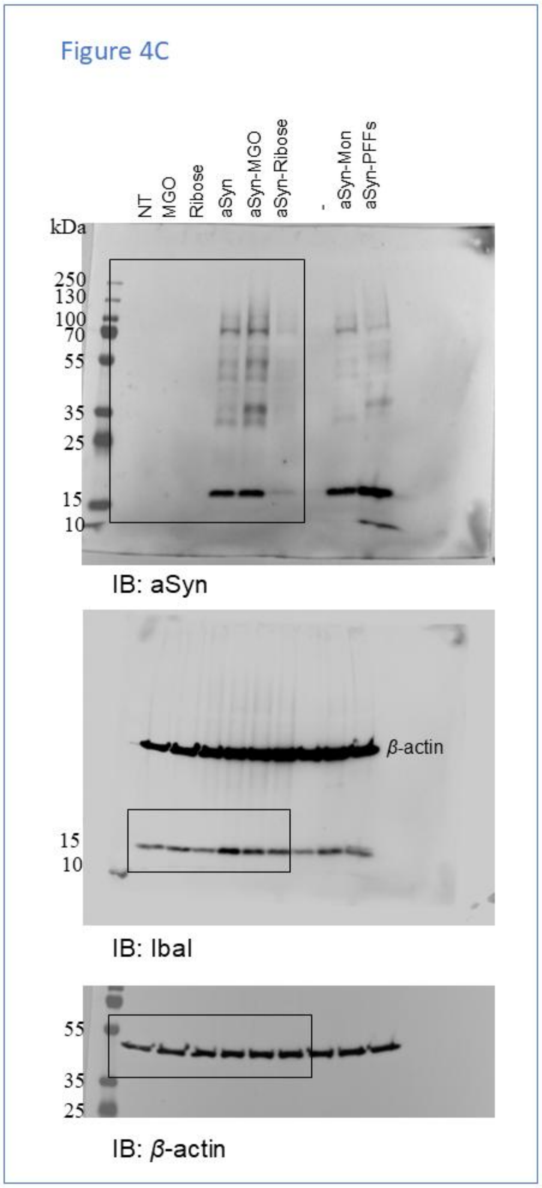

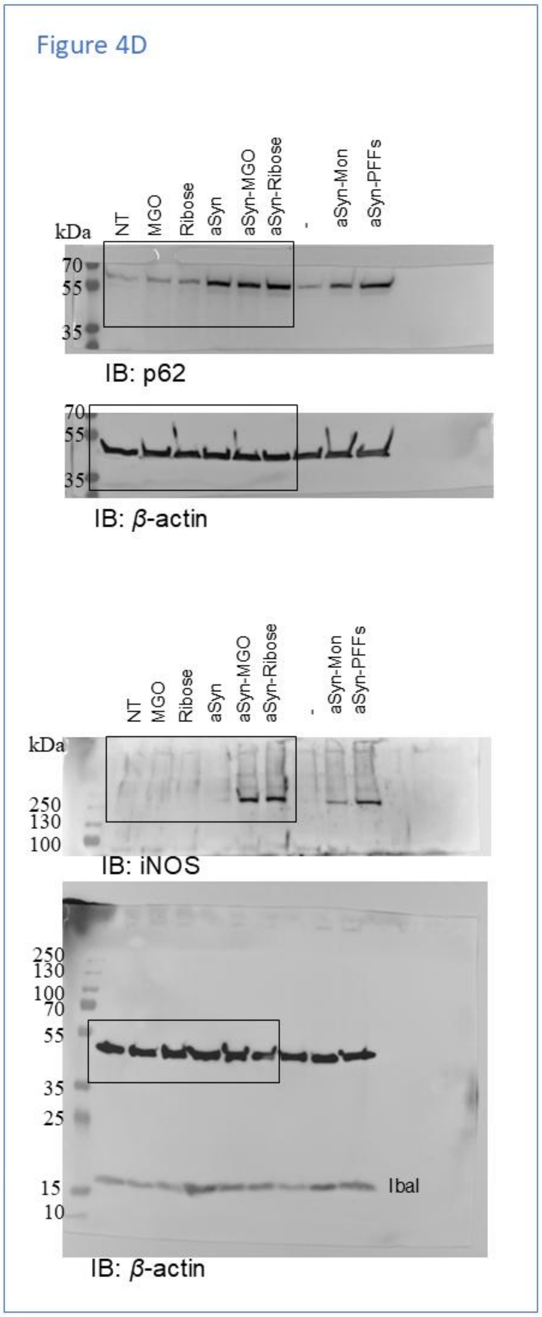

Supplementary Figure 5. Original uncropped and unprocessed WB scans for main figures are provided. Specific details are provided below the respective immunoblots in Supplementary Figure 5. In Figure 3A, 4C and 4D, data grouping involved lane removal from blots, indicated by dividing lines on immunoblots and squares highlighting the retained lanes in the corresponding uncropped blots (Supplementary Figure 5).

Supplementary References

1 Chegão, A. *et al.* Glycation modulates glutamatergic signaling and exacerbates Parkinson’s disease-like phenotypes. *npj Parkinson's Disease 2022 8:1* **8**, 1-22, doi:10.1038/s41531-022-00314-x (2022).
